# Translating and testing the Alberta context tool for use among nurses in Swedish elder care

**DOI:** 10.1186/1472-6963-13-68

**Published:** 2013-02-19

**Authors:** Ann Catrine Eldh, Anna Ehrenberg, Janet E Squires, Carole A Estabrooks, Lars Wallin

**Affiliations:** 1 Karolinska Institute, Department of Neurobiology, Care Sciences and Society, Stockholm, Sweden; 2 Dalarna University, School of Health and Social Studies, Falun, Sweden; 3 University of Ottawa, School of Nursing, Faculty of Health Sciences, Ottawa, Canada; 4 Ottawa Hospital Research Institute, Clinical Epidemiology Program, Ottawa, Canada; 5 University of Alberta, Faculty of Nursing, Knowledge Utilization Studies Program, Edmonton, Canada; 6 CRU, Karolinska University Hospital Solna, Eugeniahemmet T4:02, SE17176, Stockholm, Sweden

**Keywords:** Questionnaire, Translation, Validity, Health care context, Research utilization, Nursing

## Abstract

**Background:**

There is emerging evidence that context is important for successful transfer of research knowledge into health care practice. The Alberta Context Tool (ACT) is a Canadian developed research-based instrument that assesses 10 modifiable concepts of organizational context considered important for health care professionals’ use of evidence. Swedish and Canadian health care have similarities in terms of organisational and professional aspects, suggesting that the ACT could be used for measuring context in Sweden. This paper reports on the translation of the ACT to Swedish and a testing of preliminary aspects of its validity, acceptability and reliability in Swedish elder care.

**Methods:**

The ACT was translated into Swedish and back-translated into English before being pilot tested in ten elder care facilities for response processes validity, acceptability and reliability (Cronbach’s alpha). Subsequently, further modification was performed.

**Results:**

In the pilot test, the nurses found the questions easy to respond to (52%) and relevant (65%), yet the questions’ clarity were mainly considered ‘neither clear nor unclear’ (52%). Missing data varied between 0 (0%) and 19 (12%) per item, the most common being 1 missing case per item (15 items). Internal consistency (Cronbach’s Alpha > .70) was reached for 5 out of 8 contextual concepts. Translation and back translation identified 21 linguistic- and semantic related issues and 3 context related deviations, resolved by developers and translators.

**Conclusion:**

Modifying an instrument is a detailed process, requiring time and consideration of the linguistic and semantic aspects of the instrument, and understanding of the context where the instrument was developed and where it is to be applied. A team, including the instrument’s developers, translators, and researchers is necessary to ensure a valid translation. This study suggests preliminary validity, reliability and acceptability evidence for the ACT when used with nurses in Swedish elder care.

## Background

In recent decades, understanding of what influences implementation of evidence based knowledge in health care has increased. Among other influences, contextual factors are important [1]. Organizational context includes both observable aspects, such as the physical environment and availability of information resources, and underlying aspects, such as social interactions and management [1]. To better understand what hinders and facilitates knowledge transfer, context needs to be assessed [2]. Apart from more generic instruments on employees’ experience of work context (such as; Situational Outlook Questionnaire (SOQ) [3], Quality-Work-Competence (QWC) [4], and The revised Nursing Work Index (NWI) [5]) there are few instruments available particularly for measuring context in relation to knowledge transfer in health care settings. When this study was initiated we recognised three instruments designated for this specific purpose: the Context Assessment Index (CAI) [6], the Organizational readiness to Change Assessment (ORCA) [7], and the Alberta Context Tool (ACT) [8]. At that time point, CAI was being tested in Sweden, resulting in a proposed need for further refinement [9], while ORCA and ACT had not previously been tested in Sweden. The ACT appeared to have promising properties for use in Swedish health care settings, considering similarities in health care organisation and professional practice in Canada and Sweden. Further, the ACT was being considered for a international study on implementation of evidence in elder care, involving both Sweden and Canada [10].

The Alberta Context Tool was designed to measure modifiable aspects of organizational context in health care settings. It is administered to individuals (i.e., health care staff) to elicit their perceptions of context at the care unit and/or facility level, depending on the context of care delivery. For nurses, this level is frequently the patient or resident care unit. The ACT was developed by Estabrooks and colleagues in Canada and consists of a series of items representing 10 modifiable contextual concepts: (1) leadership, (2) culture, (3) evaluation, (4) social capital, (5) structural and electronic resources, (6) formal interactions, (7) informal interactions, (8) organizational slack – staffing, (9) organizational slack – space, and (10) organizational slack – time [8].

The theoretical framing for the ACT was the Promoting Action on Research Implementation in Health Services (PARIHS) framework [1], and related literature in the fields of organizational science, research implementation, and knowledge translation [11-13]. The ACT exists in 3 versions (acute care, long-term care and home care), each with multiple forms for different target groups (health care assistants/aides (HCA); nurses, i.e., licensed practical nurses (LPN) and registered nurses (RN); allied health professionals; physicians; practice specialists (e.g. educators), and care managers). Further, the ACT is accompanied by a set of demographic questions regarding sex, age, education, professional experience and working hours. Scores obtained using the ACT with nurses and HCA’s in paediatric care and residential long-term care facilities respectively, have demonstrated acceptability, reliability, and construct validity [14,15]. Further, the ACT has been applied in studies on knowledge transfer in health care in Canada and Australia [16-19].

Swedish and Canadian health care have similarities with regards to a variety of aspects, such as political governance and health care financing [20], health care policies and legislation, and health care professionals’ education and staff perceptions, e.g., with regards to the concept of research utilization among nurses [21]. As a result, the need for an instrument to measure organizational context in knowledge transfer research in Sweden prompted a decision to translate ACT to Swedish, rather than constructing a new instrument. Also, with the similarities between the countries, the possibility for cross-country comparisons was of interest.

Translation of an instrument should consider linguistic, semantic and contextual aspects, aiming to create a valid instrument for the new setting [22]. These aspects refer to a) words and grammar (linguistics), b) concepts (semantics), and c) the context where the instrument was developed and shall be applied, respectively. The linguistic and semantic aspects require consideration of language; For example, according to Ogden and Richard’s [23] theory on language meaning, there is an indirect relation between a term and a phenomenon by the use of a thought or idea, presented as a concept. When sharing words, as in speaking, listening or reading, the reference to the phenomenon needs to be common for the utterer and the receiver, provided by the shared idea (the concept) linking the word to the phenomenon. Thus, when translating words, we need to make sure not only that we use equal terms but also that we have a common idea of the phenomenon, manifested by the concept. In order for a valid process and outcome, back translation and inclusion of experts and the original instrument developers in the process are important [22], to manage potential semantic and contextual issues. Since all three aspects need to be considered, i.e. linguistic, semantic and contextual, translation of an instrument signifies a complex and demanding undertaking [24]. This paper reports on the translation of the ACT to Swedish, including linguistic, semantic and contextual aspects, and a testing of preliminary aspects of its validity as well as acceptability and reliability in elder care.

## Methods

### Design

Translation and pilot testing of the ACT, including assessment for validity (in terms of response processes), acceptability, and reliability [25], using both quantitative and qualitative methods.

### Procedures

**Figure 1:**
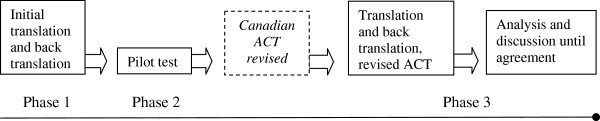
Overview of the translation and validation process.

As illustrated in Figure 1, the process of translating and pilot testing the ACT comprised three phases, which started in early 2009 and finished in spring 2010. This represents a modified version of the process outlined by Streiner and Norman [26].

#### Phase 1 - initial translation and back translation

Translation of ACT (long-term care – nurse) from Canadian English to Swedish was performed by two Swedish researchers fluent in English. A back-translation was performed by a professional language reviewer, fluent in both languages. Subsequently, two LPNs and two RNs working in one Swedish elder care facility examined the translated questionnaire to see if it made sense and to complete the tool. The researcher met with them at their workplace, where they individually responded to the questionnaire and provided immediate feedback on any questions or obstacles that they perceived while responding to the questions.

#### Phase 2 - pilot test

The ACT was pilot tested in 10 Swedish elder care facilities, for response processes validity, acceptability, and reliability. Concurrently to the Swedish pilot test, a modified version of the ACT for long-term care nurses was developed and tested in Canada (in English) and presented to the Swedish research team [8].

#### Phase 3 - translation and back translation of the modified ACT

The modified version of the ACT was translated, incorporating the experiences and findings from the initial translation and back translation, and the pilot test. Further, the modified ACT was back translated from Swedish to English by a professional language reviewer. The reliability findings (Cronbach’s Alpha) of the pilot test were reanalysed according to the item structure presented in the modified ACT. In addition, the Swedish researchers performed linguistic, semantic, and contextual analysis on the modified translated ACT. Consensus discussions between the Canadian developers and the Swedish researchers also occurred and continued until both parties reached full consensus in all aspects of the translation.

### The Alberta context tool

The long-term care nurse version of the ACT was developed to assess professional nurses’ (RNs and LPNs) perceptions of context. In Sweden, long-term care is referred to as residential care for the elderly (i.e., elder care), including facilities corresponding to nursing homes.

The initial version of ACT applied in Phases 1 and 2 of this study comprised 59 statements, organized in 10 contextual concepts. Each statement was scored on a 5 point Likert (strongly disagree to strongly agree) or frequency (never to almost always, with a not available option where appropriate) response scale. For Phase 3, the ACT for long-term care was modified by the Canadian developers. Changes included three items being transferred between concepts, renaming of contextual concepts, additional guidance on how to respond to items, and layout changes to guide respondents to the appropriate follow-up items [17]. Wording of items and scale responses between this version (Phase 3) and that used in the pilot test (Phase 2) were the same with the following minor exceptions: (1) two changes of words to clarify that the questions respectively were considering the respondent’s work unit, (2) one alteration of order of words and (3) one additional phrase to clarify a profession (i.e. ‘health care aides or health care assistants’).

### Pilot test

#### Setting and sample

The pilot test was conducted in two Swedish municipalities chosen by convenience in order to include about 300 respondents working in elder care. The municipalities were representative of other areas in Sweden, thus including both urban and rural areas, and included 10 residential elder care facilities. The pilot study was performed with nurses, including RNs and LPNs.

The pilot test population consisted of all 36 RNs and 252 LPNs employed at the 10 elder care facilities, excluding nurses who had worked less than three months at the units prior to the study and nurses on leave during the data collection period. An overview of the pilot test population, respondents and demographics is provided in Table 1.

**Table 1:** Pilot test population, respondents and demographics

**Profession**	**Population**	**Respondents**	**Women**	**Men**	**Years in profession**	**Years in working place**
	n (%)	n (%)	n (%)	n (%)	m (SD)	m (SD)
Registered nurses	36 (13)	24 (15)	22 (14)	2 (33)	18.0 (13.7)	6.5 (7.5)
Licensed practical nurses	252 (87)	131 (82)	127 (83)	4 (67)	15.9 (10.3)	8.2 (8.3)
Missing data		4 (3)	4 (3)			
Total	288 (100)	159 (100)	153 (100)	6 (100)		

#### Pilot test procedure

The managers provided verbal and written information to the nurses on their units about the study, and distributed the ACT questionnaires along with envelopes with prepaid postage for return of their responses. Reminders were distributed twice via the managers: two weeks and one week prior to the last day for responding.

Three questions to assess the respondents’ experience of completing the ACT (i.e., response processes validity) were added to the questionnaire, following the ACT items. Response processes validity refers to empirical evidence of the fit between the concept under study (dimensions of context) and the responses given by respondents on the item(s) developed to measure the concept [25]. Response processes validity evidence can come in a variety of forms but is most often derived from observations or interviews employed to determine if an individual’s behavior or verbal explanation(s) are congruent with their responses to an instrument item/question [27]. The three questions addressed: 1) ease in responding to the ACT items, 2) relevance of the ACT items, and 3) clarity of the ACT items. Each question was phrased as a statement, scored by means of a 5-point Likert scale: ‘Strongly disagree’ to ‘Strongly agree’. Space for additional free text comments was also provided.

### Data analysis

#### Phase 1 - translation and back-translation

The Phase 1 translation and back-translation was analysed for linguistic, semantic and contextual equivalence between the Swedish translation and the original ACT by both the Swedish researchers and the Canadian developers [28]. The assessment for linguistics and semantics was done using a scoring of each item as either ‘Equal wording or slight differences but conceptually OK’ or ‘Deviation in wording, potentially changed meaning conceptually’, with additional comments in free text capturing contextual (country) issues.

#### Phase 2 - pilot test

In Phase 2, response processes validity was assessed by examining respondents’: (1) responses to the three questions added to the questionnaire on ease in responding, relevance, and clarity of the ACT, and (2) additional comments. Responses to the three added questions were analyzed with descriptive statistics using Statistical Package for the Social Sciences (SPSS 19). The additional comments in free text were transcribed verbatim and analyzed using content analysis [29]; meaning units were condensed and categorized based on what the text said, i.e., the manifest content. Further, acceptability was examined by missing data frequencies and reliability was examined using internal consistency (Cronbach’s Alpha) coefficients. Coefficients can range from 0 to 1; a coefficient of 0.70 is considered acceptable for newly developed scales while 0.80 or higher is preferred, indicating that the items may be used interchangeably [30].

#### Phase 3 - translation and back translation of the modified ACT

In Phase 3, the modified ACT was translated including the experiences from the phase 1 translation and back translation and the phase 2 pilot test. Again, the modified ACT was back translated, followed by analyses of the translation and back-translation by the Swedish researchers and the Canadian developers, respectively. Discussion continued until agreement was reached, requiring the translation to include the most accurate phrasing in Swedish to correspond to the Canadian original text and its meaning. This required use of Swedish terminology and grammar that are appropriate in terms of everyday language in Swedish elder care.

### Ethics

Permission to translate and use the ACT was obtained from its developer and copyright holder (Estabrooks). Ethical approval for the pilot test was obtained from the Dalarna University Research Ethics Committee, Sweden. Operational approval was obtained from managers of the care units for elderly involved in the pilot study. The nurses who participated in the pilot study were informed in writing about the voluntary nature of participation and that decision to participate would not impact on their working conditions. A returned questionnaire implied consent.

## Results

### Response processes

Response processes validity evidence came from two samples: four nurses (two LPNs and two RNs) who examined the ACT in Phase 1 (translation and back-translation) and the 159 nurses participating in Phase 2 (the pilot study). The four nurses examining the ACT prior to the pilot test indicated a need for minor revision of language only: one referential error was corrected and one term was exchanged. In the pilot study, 159 nurses (55%) responded to the ACT; over half (52%) of the responding nurses indicated that the ACT items were easy to respond to, indicating feasibility of their use in Swedish elder care. Additionally, 65% of the nurses indicated that the ACT items were relevant to their setting and work. However, clarity of the ACT items was equivocal with over half (52%) of the nurses suggesting the translated ACT items were ‘neither clear nor unclear’ (see Table 2).

**Table 2:** Nurses’ answers regarding response processes

	**Agree, n (%)**	**Neither agree nor disagree, n (%)**	**Disagree, n (%)**	**Total, n (%)**
Questions easy to respond to	RNs 7 (30)	RNs 13 (57)	RNs 3 (13)	23 (96)
	LPNs 72 (56)	LPNs 45 (35)	LPNs 11 (9)	128 (98)
Questions relevant	RNs 14 (58)	RNs 9 (38)	RNs 1 (4)	24 (100)
	LPNs 80 (67)	LPNs 39 (32)	LPNs 1 (<1)	120 (92)
Questions clear	RNs 9 (38)	RNs 7 (29)	RNs 8 (33)	24 (100)
	LPNs 46 (38)	LPNs 68 (57)	LPNs 6 (5)	120 (92)

Additional comments were provided by 21% of the respondents (19 LPNs and 15 RNs), accounting for 20 and 21 comments respectively. The LPNs’ comments primarily referred to the ACT items being complicated and hard to understand (n = 8), including unfamiliar words (n = 3). RN comments mainly concerned the ambiguity of concepts, such as: working group, care team and work place (n = 4), and of response scales (n = 4), while 6 RNs commented on altogether 9 items they considered unclear and why this was the case. Unclarity of the items is exemplified by the following quotes.

‘What is meant by productivity (in the item ‘My organization effectively *balances* best practice and productivity’)?’ (Italics as in ACT).

‘Individuals, who are they?’ (Re the item ‘Individuals who participate in group activities are valued by others in the group’).

Both LPNs and RNs commented that there were items not relevant for the organization (n = 2 and 3, respectively). Further, one LPN perceived that the questionnaire contained too many questions and took too long to respond to and one RN pointed out the difficulty in responding due to limited experience of the particular work place.

### Acceptability

Acceptability was assessed using missing data frequencies. Missing data varied between 0 (0%) and 19 (12%) cases (nurses) per item, the most common being 1 missing case per item occurring in15 items. Three ACT items had 10 or more missing cases : (1) attendance at ‘In-services/workshops/courses’ (n = 10 missing cases); (2) attendance at ‘continuing education held outside the elder care facility’ (n = 10 missing cases); and, (3) frequency of ‘residential care related discussions with health care assistants’ (n = 19 missing cases).

### Reliability

The findings of the pilot study showed acceptable internal consistency (Cronbach’s Alpha > .70) [31] for most contextual concepts, as presented in Table 3. However, the contextual concept ‘Informal interactions’ showed lower internal consistency (Cronbach’s Alpha 0.56), along with ‘Culture’ and ‘Social capital’ (Cronbach’s Alpha 0.63 and 0.68, respectively).

**Table 3:** Internal consistency for eight contextual concepts of ACT in elder care

**ACT- contextual concepts**	**Cronbach’s alpha**	
	**Pilot study**	**Pilot study data analysed by the modified ACT**
Leadership	0.87 (n = 149)	No change to contextual concept
Culture	0.63 (n = 148)	No change to contextual concept^i^
Evaluation	0.87 (n = 147)	No change to contextual concept
Informal interactions	0.56 (n = 134)	0.63
Formal interactions	0.73 (n = 153)	0.80
Social capital	0.68 (n = 149)	No change to contextual concept
Structural and electronic resources^ii^	0.77 (n = 118)	0.77
Organizational slack (staffing, space, time)^iii^	0.87 (n = 133)	No change to contextual concept

^i^ In an analysis excluding the item with a translation error, the pilot test data reached a Cronbach’s Alpha of 0.71 for the contextual concept Culture.

^ii^ The response alternative ‘Not available’, presented for items regarding structural and electronic resources, was treated as ‘Never’ in the pilot test analysis, as a not available resource could not be used.

^iii^ In the revised ACT, organizational slack is derived as three separate concepts: staffing, space, and time.

In phase 3, the data from the pilot test was applied for reanalysis to the structure of the modified version of the ACT. Based on psychometric analyses, this meant that two items were moved from the contextual concept ‘Formal interactions’ to ‘Informal interactions’ and one item on attendance of ‘In-services/workshops/courses in your facility’ was moved from the contextual concept ‘Structural and electronic resources’ to ‘Formal interactions’[8]. In our reanalysis of these three concepts, the following Cronbach’s Alpha coefficients were determined: ‘Informal interactions’ (Alpha = 0.63, increase from previous value of 0.56); ‘Formal interactions’ (Alpha = 0.80, increase from previous value of 0.73); and, ‘Structural and electronic resources’ (Alpha = 0.77, unchanged from previous value), see Table 3.

### Translation

The back translation of the ACT used for the pilot test included 57 items. The Swedish researchers and the Canadian developers identified 30 discrepancies between the original (English) and Swedish translated forms that required discussion and consensus (see Table 4).

**Table 4:** Identified linguistic and semantic issues in the phase 1 translation and back translation of the ACT

**Team (Swe for Sweden, Ca for Canada)**	**Equal wording or slight differences but conceptually OK**	**Questioned if equal in wording or with slight differences/need for minor change**	**Deviating**
	**No of items (%)**		
Swe	50 (88)	3	2
Ca	28 (49)	7	18

During the translation, back translation and consensus process, linguistic and semantic as well as contextual (country-specific) challenges were encountered. Linguistic challenges included English words not existing in Swedish (e.g. ‘champions’ in the sentence ‘Someone who champions research in practice’, substituted by ‘advocates’), words not being common language (e.g. ‘family conferences’, substituted by ‘care planning with family’), and Swedish homonyms (i.e., words with the same pronunciation and spelling but different meaning) and synonyms. The main semantic challenges were to find terms that captured the essence of the ACT concepts in English, yet being expressed in everyday Swedish language. For example, ‘mentors’ in the ACT item ‘actively mentors or coaches performance of others’ may be translated to three possible words in Swedish (equivalent to ‘leads’, ‘supervises’ or ‘guides’). The word was translated as ‘guides’ in Swedish, but ‘guides’ was then back translated to English as ‘leads’, which carries a different meaning from ‘mentors’ in the English language. Other challenges included translating English terms and phrases that do not exist in Swedish. For example, ‘the best day’ in the ACT item ‘we have enough staff to make sure the residents have the best day’ does not have a corresponding expression in Swedish. In the end, this challenge was overcome by using ‘we have enough staff to make sure residents have the best situation achievable’. Finally, contextual (country specific) challenges centred around acknowledging the structure and various functions in Canadian health care, identifying equivalents (or if not possible, nearly equivalents) in Swedish health care. The deviations between countries concerned non-existing roles, and organisation of care including work shift hours, and title of managers. An example was managing the translation of ‘nurse practitioner’, which was a part of an item containing several options (nurse practitioner/clinical instructor /lecturer/specialist nurse). Nurse practitioner is a profession common in Canada which does not exist in Swedish health care. Therefore, this term was excluded, leaving the item with the options ‘clinical instructor /lecturer/specialist nurse’. Further, the analysis recognized one item within the contextual concept ‘Culture’ as being misinterpreted in the translation process; in the pilot study version the item ‘a supportive work group’ had been translated to ‘support work group’, the latter indicating a supervised group rather than ones work team being supportive. When this item was excluded from the pilot test analysis, acceptable homogeneity of the contextual concept Culture was reached (Cronbach’s alpha 0.71, rather than 0.63). Linguistics or semantics did not explain lower internal inconsistency values for other ACT concepts.

When the modified long-term care version of ACT presented by the Canadian developers was translated to Swedish in Phase 3, experiences of the previous translation and back translation, and the pilot test were considered. After the translation and back-translation of the new version, 101 translated and back translated paragraphs (including headings, demographics, stems, and items) were shared with the Canadian developers. Altogether, 21 linguistic- and semantic-related issues and 3 context (country) related deviations were identified and discussed, as presented in Table 5. With two cycles of correspondence between the Swedish researchers and the ACT (Canadian) developers, consensus was achieved and a final Swedish version of the ACT Long-Term Care Nurse form was approved. The final approved form conformed to the Swedish context and language (as in linguistics) as well as to the semantic meaning of the ACT concepts in the original Canadian form.

**Table 5:** Linguistic, semantic and context related deviations in final translation of the ACT

**Description of translational issue (Swe for Sweden, Ca for Canada)**	**Type of issue**	**Discussion (Swe for Sweden, Ca for Canada)**	**Final decision**
Swe: Suggest calling the team ‘nursing team’ in Swedish since there are no other teams in long term care	Context	Ca: The survey includes questions about interactions with physicians, therapists, etc.	‘Nursing’ removed
Ca: ‘with respect to’ not equivalent to ‘starting point’	Raised as semantic, revealed as linguistic	Swe: the word used for ‘starting point’ is equivalent to ‘think of’	Agreed on ‘think of’
Swe: ‘role’ has two meanings, the equivalent of function is the closest	Semantic	Ca: the item is mainly about ensuring the questionnaire is filled out by the target group for the survey and not another health care provider	Agreed on ‘function’
Ca: Number of shifts and number of hours per shift differs between Ca and Swe	Context	Swe: Standard shifts are morning (7.00 -8.00, finishing 13.00 - 16.00), afternoon (start at 11–15, end at 19–21) and night shift	Agreed to country specific groups
Swe: One word equals manager or head or boss	Linguistic	Ca: Manager needs to be specified as home manager	Agreed
Ca: ‘Difficult to hear’ needs a better translation	Linguistic	Swe: Suggest equivalent to ‘unpleasant to hear’	Agreed
Swe: Leave out the word ‘acknowledges’ since no Swedish equivalent works in the context	Linguistic	Ca: This does not impact on meaning	Agreed
Ca: There is a difference between solving and resolving	Raised as semantic, revealed as linguistic	Swe: There is one word only, meaning solving and resolving	Agreed to use suggested word
Swe: There is no equivalent to ‘best practice’	Semantic	Ca: Suggested ‘best possible care’ all right	Agreed
Ca: Having two words separated by a slash makes items double barrelled	Linguistic	Swe: Suggest ‘team’ rather than ‘group’ to equal team and ‘relatives’ to equal family	Agreed
Ca: Aim for the notion of ‘last’ typical month	Semantic	Swe: Suggest we use ‘last ordinary’	Agreed
Ca: ‘attended’ is different from ‘participated’ in English	Raised as semantic, revealed as linguistic	Swe: There is one word meaning both ‘participated’ and ‘attended’	Agreed
Swe: There is no equivalent to respiratory therapist in Sweden	Context	Ca: OK to exclude	Agreed
Swe: ‘Bedside teaching’ does not exist in elder care but suggest ‘Informal teaching in direct nursing care situations’	Linguistic	Ca: OK	Agreed
Ca: ‘Routinely’ needs an equivalent	Linguistic	Swe: No exact word works in this context but suggest using ‘generally’	Agreed
Ca: Does ‘those assigned responsible’ equate to ‘positions of authority’?	Semantic	Swe: Yes	Agreed
Ca: ‘comfortable’ needs an equivalent	Linguistic	Swe: ‘Comfortable’ can be used even if it means applying informal language in the item	Agreed
Swe: ‘team exchanges’ needs to be specified as ‘information exchange’ to clarify that it’s not just any exchange (e.g., recipes)	Semantic	Ca: OK	Agreed
Swe: The word for library is better understood if in definite form or to write it in the plural	Linguistic	Ca: Please use definite form	Agreed
Ca: Please provide equivalent to ‘best day’	Semantic	Swe: ‘best situation achievable’ is the closest	Agreed
Ca: Please provide equivalent to ‘private space’	Semantic	Swe: Suggest using a word indicating both ‘separate’ and ‘private’	Agreed
Ca: Scale should range from ‘Strongly disagree’ to ‘Strongly agree’	Linguistic	Swe: Suggest we use the standard Likert scale wording applied in Swedish	Agreed
Swe: If phrasing How often do you…’ in Swedish in this context, it reads ‘How often do you have…’	Linguistic	Ca: OK	Agreed
Swe: Does ‘care of plan for resident’ equal ‘resident’s care plan’?	Semantic	Ca: Yes it does	Agreed to translate as ‘resident’s care plan’

## Discussion

In the initiation of Phase 1, i.e. the first translation of the ACT for nurses in long term care to Swedish, the translation process felt uncomplicated by the Swedish researchers. Further, the initial responses regarding experiences of responding in a group of four nurses indicated few difficulties in understanding and responding to the instrument. However, after analysis of the Phase 1 translation and back translation, we discovered incongruence between the Canadian original and the Swedish translation that needed attention. This was further supported by the pilot test in Phase 2, where we found that a translation error (in an item in the ACT concept of culture) could explain suboptimal reliability. Further, the translation and back translation of the modified Canadian version of ACT for long term care in Phase 3 identified additional linguistic, semantic and contextual (country) issues. Our experiences emphasize the need for a careful and collaborative approach in cross-cultural translation and testing of instruments [28].

To us it was important to provide, insofar as it was possible, everyday language in the translation, yet staying true to the original meaning of the items. This required attention to semantics and extensive discussions about the meaning of a concept and exact terms used to denote it [23]. In this study, we accomplished this through continuous dialogue between the translating and testing research team and the original tool developers. An important issue that we encountered is how to handle translation when there are no equivalent terms for a concept in the translated language. For instance, English has a large number of synonyms [32], compared to Swedish. This is possibly influenced by two factors: First, even though both English and Swedish have their origin in Germanic languages, English was also later influenced by the Romance languages [33], resulting in the development of a greater diversity of terms (synonyms) in the English language. Second, 326 million people speak English as their first language (compared to the 9.5 million people who speak Swedish as their first language); this too has contributed to a more rapid expansion of the English language [34]. Therefore, in instances where no equivalent Swedish terms were available for an English word or phrase, we sought consensus on whether a verbatim translation was essential. Our response processes data demonstrated that while most Swedish nurses were able to answer ACT items that contained words and phrases that were uncommon in everyday Swedish language, they largely considered them equivocal in clarity (i.e., as ‘neither clear nor unclear’). Thus, in Phase 3, we aimed for the best possible translation, recognizing the barriers of language differences, the conceptual meaning of each word, and differences in Canadian and Swedish elder care contexts.

There are several approaches to instrument translation [35]; from a forward translation only, through back translation and monolingual test to back translation in addition to mono- and bilingual tests. In this study, we settled for the mid-approach; translation and back translation in addition to testing the translated instrument in a target language sample. Even though the above process is considered both time and cost consuming, the benefit is better semantic equivalence between the source language and target language versions and the ability to test reliability and validity in the target group [35]. By sharing back translations of the initial and the modified Canadian versions of the ACT and by carrying out the subsequent analysis and dialogue, the Swedish researchers and the Canadian instrument developers were able to detect important linguistic and semantic discrepancies. An instrument such as the ACT, with 59 items, requires considerable time for dialogue and correspondence between developers and translators for a valid translation.

The context of long term care in Canada and elder care in Sweden reflects differences that needed consideration in the translation process. In the pilot test, 19 nurses did not respond to the item regarding information exchange with health care assistants (HCA). When analysing the additional comments, one nurse described that there were no HCAs in the organization, providing a possible explanation for the lack of responses to this particular item. Further, the version of ACT used in this study was designated for ‘nurses’; in Canada referring to LPNs and RNs. These two groups of nurses may have similar roles in Canadian long term care, whereas in Swedish elder care the team providing direct nursing care consists of either LPNs and HCAs, or LPNs only. RNs may be a part of the nursing team or they may have a consultant role for the nursing teams on the units, depending on how the nursing care is organized. We found this somewhat mirrored in the response processes data, where LPNs and RNs differed to some extent in how they perceived responding to the ACT, and also pointed out ambiguous words such as working group, care team and work place. Two separate questionnaires have since been developed for Swedish use with LPNs and RNs in elder care respectively, with identical items but unique demographics. This will allow for separate national (Sweden)-level analysis for RNs and LPNs while still allowing for a merged (all nurses) analysis to perform cross-country comparisons where appropriate.

Poor layout [36] or poor item order [37] can cause non-responses to particular items. In this study, there was, for example, 6% missing data on the item ‘in-service training’ in the pilot test. The heading for the item was identical to a prior instruction, where respondents were advised to respond only if given particular responses to a further prior item. Thus, respondents may have missed that they should respond to the item ‘in-service training’. As we assumed that the high number of non-responses could be an effect of poor layout in the pilot test ACT version [38], we compared the pilot test data to that of a later study employing the modified version of ACT in elder care with a similar administration procedure; in the modified version respondents were guided by arrows directing to either the follow up questions and then to the item on in-service training, or straight to ‘in-service training’. With the modified ACT, non responses for the item ‘in-service training’ was <1%. We suggest this signifies the importance of layout and design in constructing questionnaires [38,39].

## Conclusion

Through the translation and testing process described in this paper, we experienced the complexities of instrument translation and discovered the need to: (a) be sensitive to contextual (country) differences and (b) the importance of a thorough and collaborative team approach [22,28]. This study confirms that translation of an instrument requires adequate time and considerable care during the translation processes. Further, we found that including the instrument developers in the process was important and enhanced our ability to complete a sound translation and robust pilot test process. A meticulous process including translation and back translation and testing the instrument in the country where it will be used provides a deeper understanding of the linguistic, semantic and contextual aspects of the instrument, in both the original and the translated version. Further, a team process serves as a mediator for improved understanding of the context (country) where the instrument was developed and where it is being translated and tested. This, in turn, will contribute to more valid cross-country comparisons of data.

## Competing interests

The authors declare that they have no competing interests.

## Authors’ contribution

AE designed and supervised the pilot study and performed the first translation and examination along with LW. ACE performed the second translation and the qualitative analysis on translations, along with the later quantitative analysis of the pilot test. CAE and JES assessed and discussed all back translations, provided recommendations to the Swedish team re translation, and participated in consensus discussions with the Swedish researchers following each back translation to obtain a final approved translated version of the ACT. ACE drafted and revised the manuscript with contribution of all co-authors. All authors read and approved the final manuscript.

## Pre-publication history

The pre-publication history for this paper can be accessed here:

http://www.biomedcentral.com/1472-6963/13/68/prepub
